# The correlation of neutrophil-to-lymphocyte ratio with the presence and activity of myasthenia gravis

**DOI:** 10.18632/oncotarget.18546

**Published:** 2017-06-16

**Authors:** De-Hao Yang, Mei-Zi Qian, Mao-Mao Wei, Jia Li, Meng-Meng Yu, Xue-Mian Lu, Hong Yang, Hai Lin, Xiang Li, Jun-Yan Zhu, Xu Zhang

**Affiliations:** ^1^ Department of Endocrinology, The Third Affiliated Hospital of Wenzhou Medical University, Wenzhou 325000, China; ^2^ Department of Anesthesiology, The First Affiliated Hospital of Wenzhou Medical University, Wenzhou 325000, China; ^3^ School of the First Clinical Medical Sciences, Wenzhou Medical University, Wenzhou 325000, China; ^4^ Department of Neurology, The First Affiliated Hospital of Wenzhou Medical University, Wenzhou 325000, China; ^5^ Institute of Diagnostic and Interventional Radiology, Shanghai Jiao Tong University Affiliated Sixth People's Hospital, Shanghai 200233, China

**Keywords:** neutrophil-to-lymphocyte ratio, myasthenia gravis, disease activity, new predictor, MGFA

## Abstract

Though the pathogenesis of myasthenia gravis (MG) is not fully understood, the role of inflammation has been well appreciated in the development of MG. We aimed to investigate the role of neutrophil-to-lymphocyte ratio (NLR) in MG patients and the relationship between the NLR and the activity of the disease. A total number of 172 MG patients and 207 healthy controls (HC) were enrolled in this study. The MG patients were divided into tertiles according to NLR (low NLR < 1.58, *n* = 57; intermediate NLR 1.58–2.33, *n* = 57 and high NLR > 2.33, *n* = 58). The disease activity assessment was performed according to the standard criteria established by the Myasthenia Gravis Foundation of America. Patients with MG had significantly higher NLR when compared with the HC group (*P* < 0.0001). The NLR levels were higher in the MG patients with severe disease activity than those with mild disease activity (*P* < 0.001), meanwhile, median NLR was statistically higher in MG patients with myasthenic crisis (MC) than those without MC (*P* < 0.001). Incidences of severe disease activity and MC were both higher in the high NLR group, compared to low and intermediate NLR groups (both *P* < 0.001). Multivariate logistic regression analysis suggested that elevated NLR was an independent predictor of severe disease activity (odds ratio = 13.201, CI% = 1.418–122.938, *P* = 0.023). These results indicate that NLR may be a simple and useful potential marker in indicating disease activity in patients with MG.

## INTRODUCTION

Myasthenia gravis (MG) is an autoimmunological inflammatory disorder characterized by fluctuating muscle weakness and abnormal fatigability [1]. The pathogenesis of MG has not been fully understood, but growing evidences have indicated that the inflammation could be a key factor of MG [2–4]. Noninvasive inflammatory markers, such as C-reactive protein (CRP), interleukin-6 (IL-6), interleukin-17 (IL-17), tumor necrosis factor-α (TNF-α), have been tested to reflex inflammatory status in MG patients [4–6]. Nevertheless, the optimal assessment for the inflammatory status of MG patients has not been developed yet.

Recently, the neutrophil-to-lymphocyte ratio (NLR), a particular white blood cells (WBC) parameter which can be easily obtained from the differential WBC count, has been identified as a new potential indicator of subclinical systemic inflammation in various diseases, including sepsis [7], vestibular neuritis [8], psoriasis [9], chronic kidney disease [10] and the like. Moreover, previous studies have shown that an elevated NLR is associated with disease activity and mortality in several diseases [11–13]. As a novel reliable indicator of systemic inflammatory status and disease activity, NLR has also been applied in estimating estimate the activity of autoimmunological diseases [14–16]. Therefore, we aimed to investigate the role of NLR in MG patients and its relationship between inflammatory response and the disease activity.

## RESULTS

### Baseline characteristics of the study subjects

Among the 379 research candidates, 172 were diagnosed as MG, 207 were HCs. The demographic characteristics of the MG patients and the HCs are elaborated in Table 1. There was not any statistically significant difference in the age or gender among two groups of the study participants. When compared with HC subjects, the MG patients, no matter male or female, had higher NLR levels (*P* < 0.001).

**Table 1:** Demographic and laboratory characteristics of MG patients and healthy controls

	MG (*n* = 172)	HC (*n* = 207)	*P* value
Age (years)	45.40 ± 17.41	43.69 ± 14.74	0.307
Gender (male, *n*.%)	71 (41.28)	94 (45.4)	0.419
WBC (× 10^9^/L)	7.19 ± 3.19	6.19 ± 1.61	< 0.001
NLR	1.94 (1.28–2.84)	1.60 (1.21–2.04)	< 0.001
Male	1.81 (1.42–2.62)	1.52 (1.20–1.95)	< 0.001
Female	2.00 (1.25–2.92)	1.64 (1.23–2.08)	< 0.001

Abbreviations: WBC, white blood cells; NLR, neutrophil-to-lymphocyte ratio.

After the evaluation of clinical laboratory data, MG patients were divided into three groups according to the values of NLR. Among these three subgroups, there were no statistical differences in terms of age, gender, body mass index (BMI), smoking habits, hypertension, diabetes mellitus, cardiopulmonary disease, RBC, hemoglobin, platelet, Tbil, Dbil, Ibil, total protein, creatinine, total cholesterol (TC), triglyceride, HDL-C, LDL-C, age of onset or thymus histology. WBC, BUN, duration of disease, and incidences of severe disease activity and myasthenic crisis of the patients in T3 group were remarkably increased when compared with those in T1 group (*P* < 0.001, *P* = 0.013, *P* = 0.046, *P* < 0.001, *P* < 0.001, respectively), conversely, albumin and UA were dramatically declined (both *P* < 0.001) (Table 2).

**Table 2:** Characteristics of patients with MG according to NLR quartiles

Variable	Total (*n* = 172)	NLR < 1.58 (*n* = 57)	1.58 ≤ NLR ≤ 2.33 (*n* = 57)	NLR > 2.33 (*n* = 58)	*P* value
Age (years)	45.40 ± 17.41	43.09 ± 17.61	43.53 ± 17.08	49.52 ± 17.07	0.085
Gender (male, n.%)	71 (41.28)	22 (38.60)	26 (45.61)	23 (39.66)	0.714
BMI (kg/m^2^)	22.49 ± 3.20	22.30 ± 2.88	22.67 ± 2.50	22.52 ± 4.06	0.827
Smoking (n.%)	29 (16.86)	12 (21.05)	11 (19.30)	6 (10.34)	0.258
Hypertension (n.%)	36 (20.93)	10 (17.54)	10 (17.54)	16 (27.59)	0.310
Diabetes mellitus (n.%)	20 (11.63)	3 (5.26)	6 (10.53)	11 (18.97)	0.069
Cardiopulmonary disease (*n*.%)	10 (5.81)	1 (1.75)	4 (7.02)	5 (8.62)	0.349
WBC (× 10^9^/L)	7.19 ± 3.19	5.60 ± 2.11	6.72 ± 2.64	9.21 ± 3.54	< 0.001
RBC (× 10^12^/L)	4.37 ± 0.50	4.36 ± 0.55	4.38 ± 0.43	4.37 ± 0.52	0.985
Hemoglobin (g/L)	132.57 ±15.25	132.21 ± 13.91	133.91 ± 15.03	131.60 ± 16.82	0.705
Platelet (× 10^9^/L)	206.70 ± 58.33	209.89 ± 57.02	207.25 ± 59.03	203.03 ± 59.71	0.818
Tbil (μmol/L)	11.33 ± 5.08	11.84 ± 5.76	11.23 ± 4.42	10.93 ± 5.03	0.622
Dbil (μmol/L)	3.94 ± 1.92	4.00 ± 2.05	3.91 ± 1.54	3.91 ± 2.15	0.962
Ibil (μmol/L)	7.39 ± 3.46	7.84 ± 3.89	7.32 ± 3.37	7.02 ± 3.09	0.436
Total protein (g/L)	69.06 ± 6.06	69.21 ± 4.38	69.91 ± 5.86	68.07 ± 7.47	0.259
Albumin (g/L)	39.68 ± 4.11	40.51 ± 4.00	40.94 ± 3.36	37.62 ± 4.16	< 0.001
BUN (mmol/L)	4.98 ± 1.62	4.80 ± 1.35	4.65 ± 1.20	5.48 ± 2.07	0.013
Creatinine (mmol/L)	58.34 ± 12.95	57.82 ± 12.22	60.77 ± 12.67	56.47 ± 13.74	0.191
UA (μmol /L)	284.26 ± 93.53	302.61 ± 83.98	306.39 ± 80.04	244.47 ± 102.86	< 0.001
TC (mmol/L)	4.69 ± 1.25	4.68 ± 0.87	4.58 ± 1.56	4.80 ± 1.25	0.645
Triglyceride (mmol/L)	1.33 ± 0.70	1.17 ± 0.54	1.40 ± 0.70	1.44 ± 0.80	0.076
HDL-C (mmol/L)	1.25 ± 0.36	1.23 ± 0.33	1.24 ± 0.41	1.28 ± 0.34	0.770
LDL-C (mmol/L)	2.79 ± 0.99	2.88 ± 0.70	2.67 ± 1.21	2.83 ± 1.00	0.497
Age of onset (years)					
< 50	100	32	35	33	0.827
≥ 50	72	25	22	25	
Duration of disease (years)					
≤ 1	121	45	42	34	0.046
> 1	51	12	15	24	
Thymus histology					
Non-thymoma	108	35	39	34	0.535
Thymoma	64	22	18	24	
MGFA Clinical Classification					
I + I I + III	146	56	51	39	< 0.001
IV + V	26	1	6	19	
Myasthenic crisis					
With	157	57	55	45	< 0.001
Without	15	0	2	13	

Abbreviations: NLR, neutrophil-to-lymphocyte ratio; BMI, body mass index; WBC, white blood cells; RBC, red blood cells. Tbil, total bilirubin; Dbil, direct bilirubin; Ibil, indirect bilirubin; BUN, blood urine nitrogen; UA, uric acid; TC, total cholesterol. HDL-C, high density lipoprotein-cholesterol; LDL-C, low density lipoprotein-cholesterol.

### The association of NLR with MG activity

**Figure 1:**
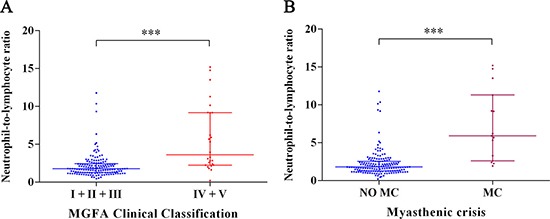
Correlation between NLR and other clinical factors in MG patients (**A**)Relationship between NLR and two subgroups according to the MGFA Clinical Classification (^***^P < 0.001). (**B**) Relationship between NLR and MC (^***^P < 0.001). Bar represents median and interquartile range.

As shown in Figure 1A, patients with higher disease activity had a higher NLR levels than those with lower disease activity. Furthermore, the levels of NLR in patients with myasthenic crisis (MC) were significantly higher in contrasted to those without MC (Figure 1B).

**Figure 2:**
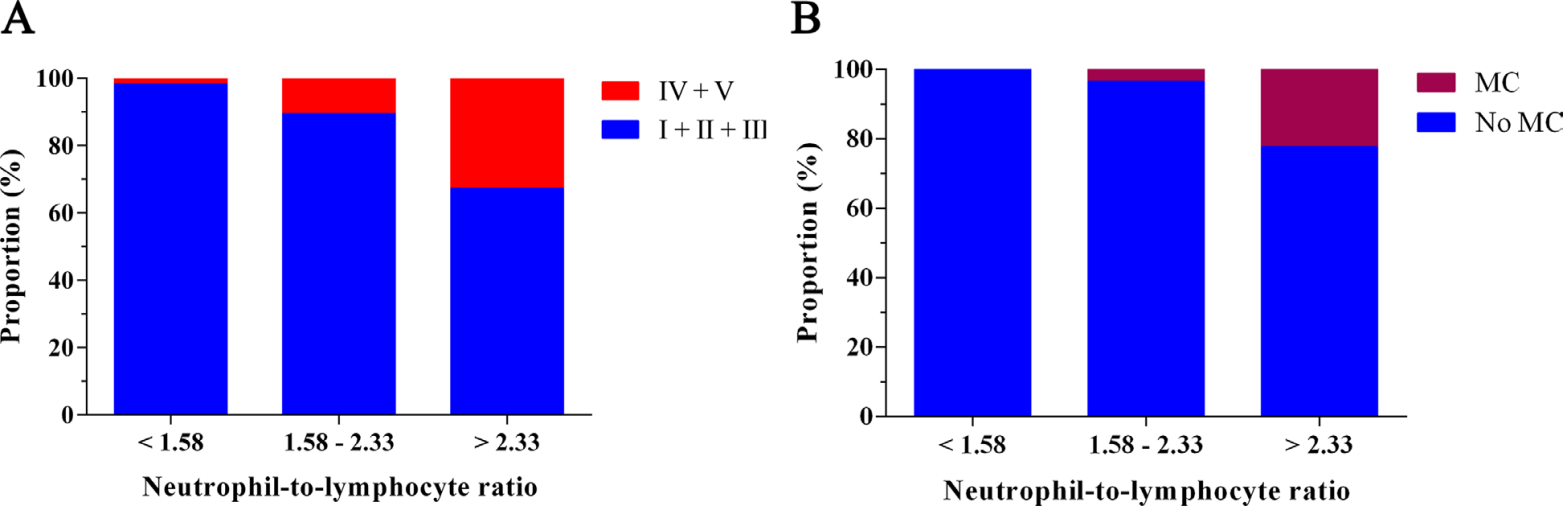
Clinical course of patients according to NLR (**A**) The disease activity of MG according to NLR. (**B**) The incidence of myasthenic crisis according to NLR.

For the purpose of clinical application, we categorized the study population according to the two cut-off values of NLR (Figure 2). In patients with a high NLR level (> 2.33), 32.76% suffered from higher disease activity. However, in patients with a moderate NLR level (1.58–2.33), 10.53% suffered from higher disease activity. In cases of patients with a low NLR level (< 1.58), only 1.75% presented with higher disease activity. Moreover, 22.41% patients with a high NLR (> 2.33) were subjected to MC while only 3.51% patients were caused to undergo MC in patients with a moderate NLR level (1.58–2.33), and none was suffered from MC in patients with a low NLR level (*P* < 0.001).

**Figure 3:**
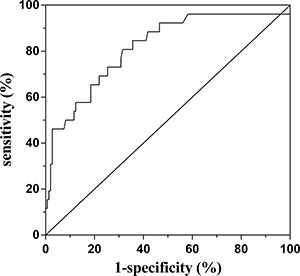
Receiver operating characteristic (ROC) curve analysis of the predictive power of the NLR for higher disease activity of MG The area under ROC curve: 0.816; 95% CI: 0.722–0.910; *P* < 0.001.

The performance of NLR levels in evaluating the disease activity with ROC analysis was cut of 2.22, 80.8% sensitivity, 68.5 % specificity, and AUC at 0.816 (95% CI: 0.722–0.910, *P* < 0.001) (Figure 3).

### Elevated NLR level is related to higher disease activity

As shown in Figure 2A, the incidences of higher disease activity of MG from T1 to T3 were 1.75%, 10.53% and 32.76%, respectively. Univariate and multivariate logistic regression analysis were performed to gain a deeper understanding of the relationship between NLR levels and the prevalence of higher disease activity. Based on univariate analysis, WBC (*P* = 0.002), Tbil (*P* = 0.047), albumin (*P* < 0.001), BUN (*P* = 0.026), creatinine (*P* = 0.003), UA (*P* < 0.001), duration of disease (*P* = 0.005) and NLR (*P* < 0.001) were all found to be significantly correlated with higher disease activity (Table 3). In model 1, differing from the subjects in T1, the odds ratio (OR) of the subjects in T3 was 27.282 (95% CI 3.505–212.350, *P* = 0.002). Adjustment for duration of disease (model 2) substantially attenuated the magnitude of the OR for higher disease activity by approximately 23.6-folds when comparing the third with the first quartile of NLR levels. Even when adjusted for WBC, Tbil, albumin, BUN, creatinine, UA, and duration of disease (Model 3), the relationship between NLR and MG activity remained significant in T3 with OR of 13.201 (95% CI: 1.418–122.938, *P* = 0.023) (Table 4). These results suggest that the subjects with higher NLR levels are more likely to develop higher disease activity of MG than individuals with lower NLR levels.

**Table 3:** Univariate logistic regression analyses of factors for severe disease activity of MG

Variables	Univariate logistic regression		
	OR	95% CI	*P* value
Age	1.018	0.993–1.043	0.160
Gender	0.717	0.300–1.716	0.455
BMI	0.972	0.848–1.113	0.676
Smoking	0.602	0.168–2.156	0.436
Hypertension	1.160	0.428–3.143	0.770
Diabetes mellitus	1.477	0.452–4.831	0.519
Cardiopulmonary disease	2.590	0.624–10.744	0.190
WBC	1.208	1.072–1.362	0.002
RBC	0.459	0.190–1.107	0.083
Hemoglobin	0.974	0.948–1.001	0.062
Platelet	0.998	0.991–1.005	0.598
Tbil	0.889	0.791–0.998	0.047
Dbil	0.735	0.535–1.008	0.056
Ibil	0.860	0.732–1.009	0.064
Total protein	1.016	0.948–1.088	0.660
Albumine	0.825	0.741–0.918	< 0.001
BUN	1.308	1.033–1.656	0.026
Creatinine	0.938	0.900–0.978	0.003
UA	0.988	0.983–0.994	< 0.001
TC	1.096	0.797–1.507	0.573
Triglyceride	0.983	0.537–1.798	0.956
HDL-C	0.416	0.110–1.571	0.196
LDL-C	1.140	0.766–1.697	0.519
Age of onset	0.847	0.360–1.992	0.703
Duration of disease	3.437	1.459–8.094	0.005
Thymus histology	1.287	0.551–3.005	0.560
NLR	1.525	1.273–1.827	< 0.001

Abbreviations: BMI, body mass index; WBC, white blood cells; RBC, red blood cells; Tbil, total bilirubin; Dbil, direct bilirubin; Ibil, indirect bilirubin; TC, total cholesterol; BUN, blood urine nitrogen; UA, uric acid; HDL-C, high density lipoprotein-cholesterol; LDL-C, low density lipoprotein-cholesterol; NLR, neutrophil-to-lymphocyte ratio.

**Table 4:** Adjusted Odds Ratio (95% Confidence Interval) for severe disease activity of MG

Variable	OR	95% CI	*P* value
Model 1			
T1	1.000	1.000–1.000	
T2	6.588	0.767–56.600	0.086
T3	27.282	3.505–212.350	0.002
Model 2			
T1	1.000	1.000–1.000	
T2	6.329	0.731–54.790	0.094
T3	23.556	2.998–185.083	0.003
Model 3			
T1	1.000	1.000–1.000	
T2	7.876	0.871–71.247	0.066
T3	13.201	1.418–122.938	0.023

Model 1 is univariate analysis. Model 2 is adjusted for duration of disease. Model 3 is adjusted for duration of disease, white blood cells, total bilirubin, albumin, blood urine nitrogen, creatinine, uric acid.

## DISCUSSION

In this study, we aimed to evaluate the association between NLR and disease activity of MG according to MGFA clinical classification. Our study showed that NLR levels were higher in patients with MG compared to those in healthy controls. Moreover, an elevated NLR was assessed to be a parameter in indicating the disease activity of MG.

To our knowledge, MG is a severe autoimmune disease characterized by loss of AChR on the postsynaptic membrane of neuromuscular junction and resulted in disordered neuromuscular transmission and muscle weakness [1]. Recently, accumulating evidences have shown that chronic inflammation response could be heavily involved in the pathogenesis of MG [6, 17, 18]. Gradolatto et al. [19] reported that chronic inflammation in the thymus of MG patients led to impaired T cell function. An evaluation index of the level of inflammatory status may help to predict which subgroup of MG patients would have a more severe disease activity.

The NLR, which represents the balance between neutrophil and lymphocyte level, has recently been proposed to be an inflammatory condition indicator of patients with various chronic inflammatory diseases [11, 20]. NLR can be easily calculated by the ratio of neutrophils to lymphocytes from routine peripheral blood test. Calculation of NLR is a pretty accessible method compared with other inflammatory cytokines, including IL-6, IL-1β, and TNF-α [21]. NLR represents a combination of these two markers, and it is superior to other leukocyte parameters (e.g., neutrophil, lymphocyte, and total leukocyte counts) because of its stability compared with other parameters which may be affected by various physiological, pathological or physical factors [22, 23]. Furthermore, Demirci et al. have shown that there is a higher NLR level in patients with multiple sclerosis (MS) than that in the healthy controls [16]. Also, Mercan et al. reported that the NLR was significantly ascended in rheumatoid arthritis patients contrast to the controls [24]. We observed significantly increased NLR in MG patients than that in healthy controls. It appears that the NLR trend to be elevated in autoimmunological diseases. A high level of NLR has been found to be correlated with the activity and poor prognosis of several diseases, including inflammatory bowel disease [14], intrahepatic cholangiocarcinoma [11] and diabetes mellitus [25]. As there is no ideal single serum inflammatory marker for predicting disease activity of MG, we stratified MG patients into three subgroups according to NLR value to explore whether NLR could be a qualified marker of the activity of MG. We determined that the presence of lifted NLR level was associated with higher disease activity via logistic regression analysis. This result suggests that the activity of MG is relevant to the degree of inflammation.

Although the detailed mechanisms behind the associations of NLR with MG remain not fully resolved, several potential mechanisms of pathogeneses have been suggested. The study has some limitations including cross-sectional design and a relatively small sample size. Our study was not designed to elucidate the mechanistic pathways which lead to higher NLR in patients with MG.

In conclusion, according to our knowledge, this is the first study which investigates the relationship between NLR, which is a newly-developing inflammatory marker, and MG and its activity. Further studies are needed to externally cross-validate our findings in a larger cohort of MG patients. Meanwhile, more laboratory experiments need to be carried out to explore the role of NLR in the pathogenesis of MG.

## MATERIALS AND METHODS

### Study population

**Figure 4:**
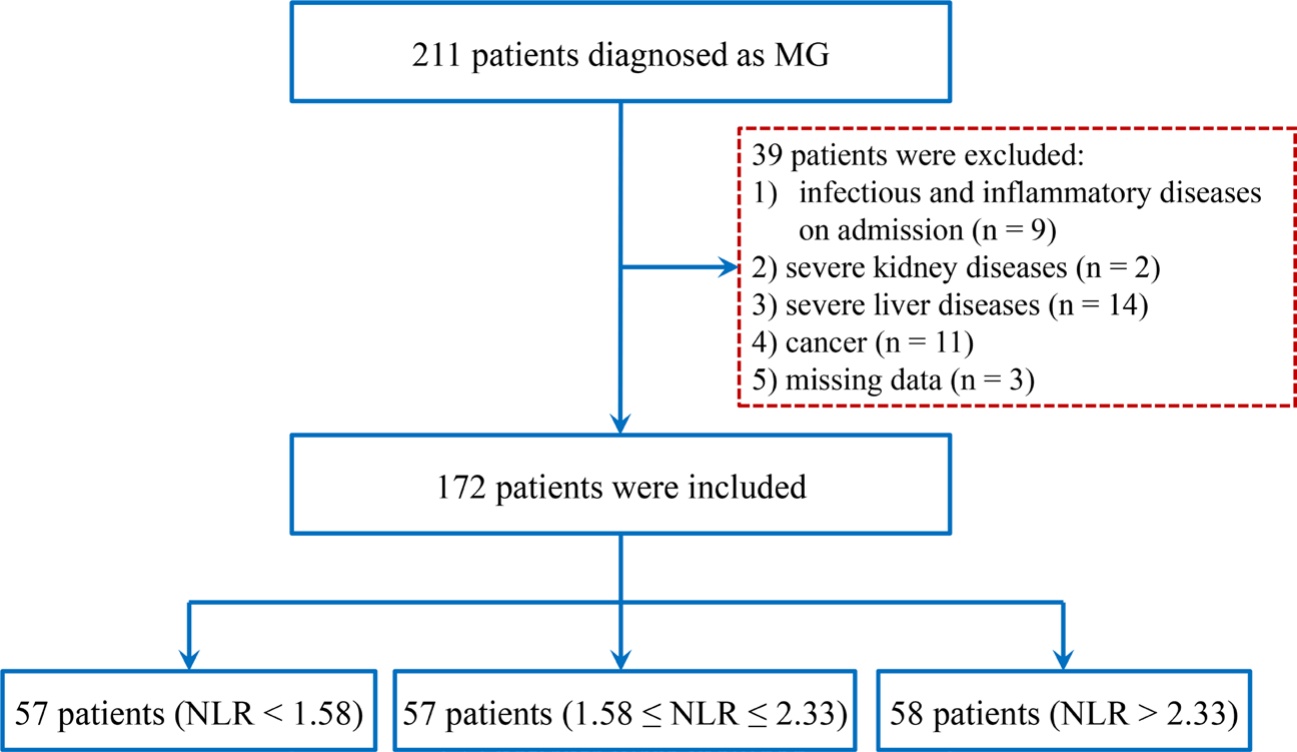
Study flow diagram

Included in the study were 172 patients who received a certain diagnosis of MG from the neurology hospitalized ward of the First Affiliated Hospital of Wenzhou Medical University and 207 age- and sex-matched healthy controls (HC) between February 2009 and March 2016 (Figure 4). Patients’ age, gender, smoking, age of onset, disease duration, thymus histology, and other medical history were all recorded for each subject. Patients with severe liver or kidney diseases, severe infectious diseases or cancer were excluded from the study. The research protocol of the study was approved by the Ethics Committee of the First Affiliated Hospital of Wenzhou Medical University.

### Diagnostic criteria

The diagnosis of MG was based on standard clinical criteria of characteristic weakness, fatigue, electrophysiology, neostigmine test and/or the presence of autoantibody against skeletal muscle acetylcholine receptors (AChRs) [26]. The activity of the disease (Table 5) was evaluated according to the Myasthenia Gravis Foundation of America (MGFA) Clinical Classification at admission [27]. Based on the classification, patients were divided into two subgroups: mild disease activity (I, II and III) and severe disease activity (IV and V).

**Table 5:** MGFA clinical classification

Class I	Any ocular muscle weakness
	May have weakness of eye closure
	All other muscle strength is normal
Class II	Mild weakness affecting other than ocular muscles
	May also have ocular muscle weakness of any severity
IIa	Predominantly affecting limb, axial muscles, or both
	May also have lesser involvement of oropharyngeal muscles
IIb	Predominantly affecting oropharyngeal, respiratory muscles, or both
	May also have lesser or equal involvement of limb, axial muscles, or both
Class III	Moderate weakness affecting other than ocular muscles
	May also have ocular muscle weakness of any severity
IIIa	Predominantly affecting limb, axial muscles, or both
	May also have lesser involvement of oropharyngeal muscles
IIIb	Predominantly affecting oropharyngeal, respiratory muscles, or both
	May also have lesser or equal involvement of limb, axial muscles, or both
Class IV	Severe weakness affecting other than ocular muscles
	May also have ocular muscle weakness of any severity
IVa	Predominantly affecting limb and/or axial muscles
	May also have lesser involvement of oropharyngeal muscles
IVb	Predominantly affecting oropharyngeal, respiratory muscles, or both
	May also have lesser or equal involvement of limb, axial muscles, or both
Class V	Defined by intubation, with or without mechanical ventilation, except when employed during routine postoperative management. The use of a feeding tube without intubation places the patient in class IVb.

### Clinical and laboratory measurements

Various variables were simultaneously obtained from the patients’ medical records, including patient demographics, laboratory and clinical tests. Blood samples were drawn by venipuncture in the morning after an overnight fast for at least 8 hours at admission. Laboratory information include WBC, red blood cells (RBC), hemoglobin, platelet, total bilirubin (Tbil), direct bilirubin (Dbil), indirect bilirubin (Ibil), albumin, blood urine nitrogen (BUN), uric acid (UA), total cholesterol (TC), triglyceride, high density lipoprotein-cholesterol (HDL-C) and low density lipoprotein-cholesterol (LDL-C). The NLR was calculated using neutrophil and lymphocyte counts. Thymus histology was presented by means of magnetic resonance imaging (MRI) or computed tomography (CT).

### Statistical analysis

In order to derive a deeper understanding of the relationship between NLR and MG activity, all subjects were classified into three groups according to the NLR level. NLR data were stratified as follows: T1: < 1.58, T2: 1.58–2.33, T3: > 2.33. All statistical analyses were performed via the statistical software Statistical Program for Social Sciences version 20.0 (SPSS Inc., Chicago, IL, USA). For continuous variables, results were expressed as mean ± standard deviation (SD), and the differences among groups were compared through one-way analysis of variance (ANOVA) or Kruskal–Wallis test. Additionally, categorical variables were presented as counts or percentages, and intergroup comparisons were analyzed through Chi-squared test. The NLR stratified according to disease activity was compared using Mann–Whitney *U* test or Kruskal–Wallis test. For the purpose of assessing the predictive performance of the NLR with respect to disease activity, the receiver operating characteristics (ROC) curves were plotted. For the sake of explaining the contribution of the variance in MG activity, multivariable models were constructed for multivariate analysis, including duration of disease, WBC, Tbil, albumin, BUN, creatinine and UA. Statistical significance was set at *P* < 0.05.
